# Hydrologic-Process-Based Soil Texture Classifications for Improved Visualization of Landscape Function

**DOI:** 10.1371/journal.pone.0131299

**Published:** 2015-06-29

**Authors:** Derek G. Groenendyk, Ty P.A. Ferré, Kelly R. Thorp, Amy K. Rice

**Affiliations:** 1 Hydrology and Water Resources, University of Arizona, Tucson, Arizona, United States of America; 2 United States Arid-Land Agricultural Research Center, United States Department of Agriculture—Agricultural Research Service, Maricopa, Arizona, United States of America; 3 Hydrologic Science and Engineering Program, Colorado School of Mines, Golden, Colorado, United States of America; NERC Centre for Ecology & Hydrology, UNITED KINGDOM

## Abstract

Soils lie at the interface between the atmosphere and the subsurface and are a key component that control ecosystem services, food production, and many other processes at the Earth’s surface. There is a long-established convention for identifying and mapping soils by texture. These readily available, georeferenced soil maps and databases are used widely in environmental sciences. Here, we show that these traditional soil classifications can be inappropriate, contributing to bias and uncertainty in applications from slope stability to water resource management. We suggest a new approach to soil classification, with a detailed example from the science of hydrology. Hydrologic simulations based on common meteorological conditions were performed using HYDRUS-1D, spanning textures identified by the United States Department of Agriculture soil texture triangle. We consider these common conditions to be: drainage from saturation, infiltration onto a drained soil, and combined infiltration and drainage events. Using a k-means clustering algorithm, we created soil classifications based on the modeled hydrologic responses of these soils. The hydrologic-process-based classifications were compared to those based on soil texture and a single hydraulic property, *K_s_*. Differences in classifications based on hydrologic response versus soil texture demonstrate that traditional soil texture classification is a poor predictor of hydrologic response. We then developed a QGIS plugin to construct soil maps combining a classification with georeferenced soil data from the Natural Resource Conservation Service. The spatial patterns of hydrologic response were more immediately informative, much simpler, and less ambiguous, for use in applications ranging from trafficability to irrigation management to flood control. The ease with which hydrologic-process-based classifications can be made, along with the improved quantitative predictions of soil responses and visualization of landscape function, suggest that hydrologic-process-based classifications should be incorporated into environmental process models and can be used to define application-specific maps of hydrologic function.

## Introduction

The organization of objects into classes is a fundamental exercise at the core of many scientific fields [1]. Taxonomy or classification of plants and animals has been traced back to the early Greeks and Romans and was later codified by Linnaeus in the Genera Plantarum in 1737 [2, 3]. There were attempts by Mendeleyev in the 1860s and Zubin in 1938 to use classification outside biology, but it was in the mid-20th century when the application of classification expanded rapidly [4]. It was advances in computer technology and formalization of classification techniques that led to modern unsupervised learning techniques such as cluster analysis [1, 4]. Classification has been used in biology for numerical taxonomy [5, 6] and cladistics [7, 8]. Social sciences has applied classification for behavior patterns and culture analysis [9, 10]. In earth sciences, it is used for earthquake detection [11] and land use patterns [12]. The medical sciences have implemented it for microorganism characterization [13] and genomics [2], engineering sciences for pattern recognition [14], and information and decision sciences for investment and economic research [15, 16]. While these applications vary widely, they share common tenets of classification: forming groups within populations based on similar characteristics or behaviors can improve understanding of interrelationships among members and can simplify the definition of characteristics for individual group members. However, there exists the possibility for misapplication of classification, leading to erroneous or even injurious associations. Ultimately, regardless of the application, effective classification requires that the application is appropriately and sufficiently linked to the basis used for clustering.

Soil classification first appeared internationally with Atterberg [17] and with the United States Department of Agriculture (USDA) [18]. In the late 1920s, the international and USDA systems were accepted formally and led to the soil classification systems in wide use today [19, 20]. The most common classifications (e.g. USDA/Food and Agricultural Organization, International Soil Science Society/International Union of Soil Sciences, Canadian, German) rely on relative fractions of soil particles of different sizes to establish soil textural class boundaries. These traditional classifications are convenient because grain size distributions can be measured relatively easily and can be estimated quickly and accurately in the field. Currently, soil texture classification is used commonly within agricultural, geotechnical, hydrological, and other related disciplines.

Previous work in the use of soil classification for hydrologic prediction has focused on manual classification of soils on the catchment scale using conceptual subsurface models. In particular, the work by Boorman, et. al. [21] was shown to improve upon standard textural classifications. In that study, the authors suggest that a more rigorous scientific approach to soil classification would be to use hydrologically relevant physical soil properties, such as hydraulic conductivity, and storage capacity in combination with a model to estimate hydrologic soil response. The authors state that this approach was not used because of the challenges of availability and coverage of physical soil property data. Our work follows on this suggestion and demonstrates the benefits of basing soil classifications on modeled hydrologic outcomes.

We focus on the use of soil texture as a proxy for soil hydraulic properties [22]. This has become increasingly common with the growth in coverage and detail of global circulation models, which require spatially distributed soil properties over large areas [23, 24]. Specifically, soil maps are used to identify boundaries within a landscape within which hydraulic properties are assumed to be constant. In addition to providing guidance for parameterization of numerical models, these soil maps are used to visualize landscape function [25]. That is, the maps guide the formulation of conceptual models of hydrologic processes that underlie model developments and guide land use, in part because of the hydrologic responses associated with different soil textures. Soil maps were developed primarily as a diagnostic tool for soil surveying and an early study by Bormann [26] suggests that using soil texture classifications may be acceptable for environmental modeling applications. The objective of this study was to examine whether soil textural classifications are useful proxies for hydraulic properties over a range of hydrologic conditions and, further, to develop an alternative approach to soil classification that can improve both quantitative analyses and visual interpretations of landscape function.

## Materials and Methods

Previous researchers have proposed the use of cluster analysis for grouping soils based on physical properties [27, 28]. Twarakavi et al. [29] showed that soil hydraulic properties map onto grain size distributions with boundaries that are similar to USDA soil textural classification. However, further research [30] showed that the responses of soils subjected to specific hydrologic conditions did not agree with soil-texture-based classifications. We extend the work of Bormann [30], introducing the concept of clustering soils based on hydrologic function. Specifically, we consider different sets of hydrologic processes for a range of soil conditions, constituting a range of hydrologic settings. We refer to the resulting soil types as hydrologic-processes-based soil texture classifications. From this basis, we quantify the ambiguity that can be introduced into hydrologic analyses when using texture-based soil classifications and demonstrate the advantages of hydrologic-process-based soil textural classifications for quantitative analyses and for generating hypotheses related to landscape-scale hydrologic function.

**Fig 1:**
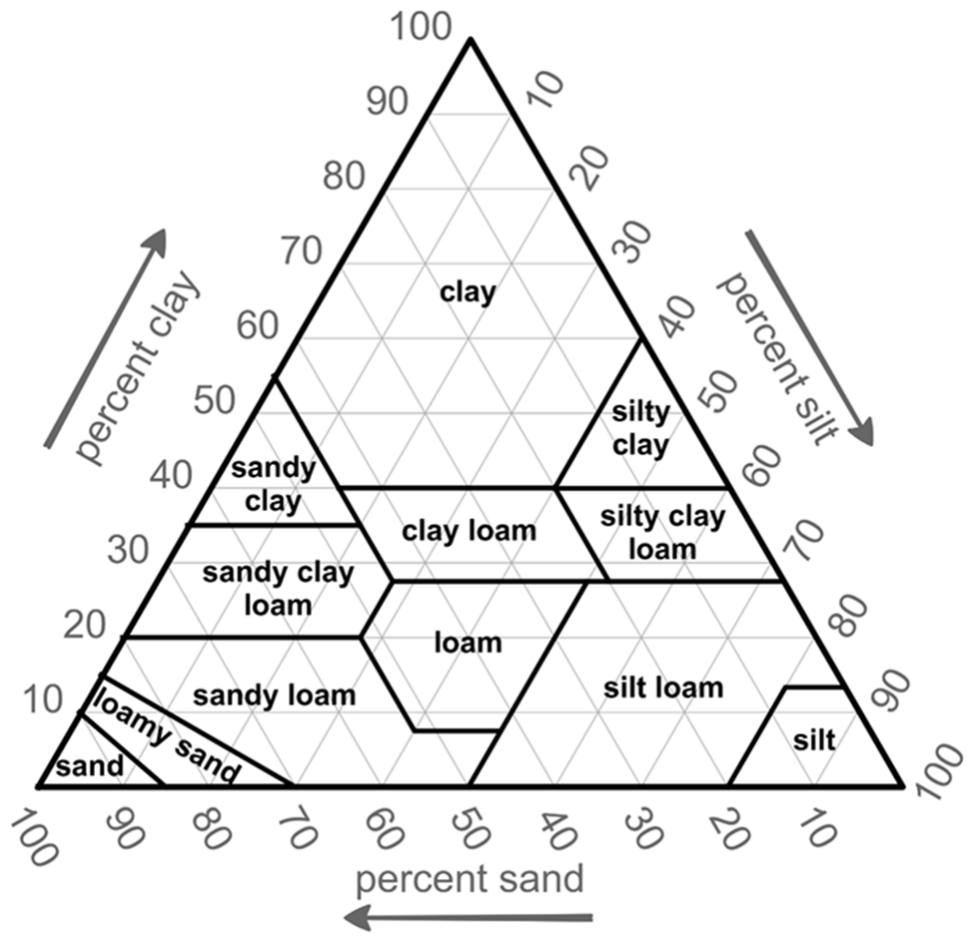
USDA Soil Texture Triangle.

For ease of comparison with the widely-used USDA soil textural classification, which was based on the mechanical limits of soil particles, we categorize soils into twelve clusters and display the cluster boundaries together with the USDA soil textural definitions on a texture triangle (Fig 1). Soils on the triangle can be identified by their respective fractions of sand, silt, and clay particles. Note that for ease of implementation, most USDA soil texture boundaries are defined by a small number of constant percentages of sand, silt or clay (straight lines, parallel to the edges of the triangle). That is, standard soils are bounded by simple polygons that allow for ready soil texture definition.

Our goal is to determine whether a range of hydrologic processes can be predicted more accurately using hydrologic-process-based soil textural classifications. To examine this, while maintaining generality, we consider three basic sets of hydrologic processes that, collectively, describe the full range of hydrologic forcings. These conditions are: drainage from a fully saturated medium; infiltration after a soil has been drained for an extended period to field capacity; and infiltration into a medium drained to field capacity followed by drainage (note that the condition preceding drainage is not full saturation throughout the profile for all soils for this case). These three conditions broadly represent subsurface hydrologic responses after a flooding event, during a rainfall event that follows a prolonged period without rain, and throughout a rainfall event onto a soil initially at field capacity immediately followed by a drying event, respectively. To demonstrate the proposed approach, we focus only on changes in water stored within a surface layer (0–30 cm depth). The target that we have chosen could represent the irrigation status in a shallow root zone, or it could represent soil conditions that control whether a field will support traffic by heavy machinery. However, depending upon the application of interest, any depth interval, hydrologically relevant state, or integration time could be specified for classification.

### Simulation Methodology

Following the approaches of Twarakavi et al. [29] and Bormann [30], we used an extensive database of soil properties compiled into ROSETTA, a neural-network based pedotransfer function [31], to relate sand, silt, and clay fractions to parameters in the van Genuchten [32] and Mualem [33] hydraulic relationships. These relationships describe the dependence of the volumetric water content on soil water pressure and the dependence of the soil hydraulic conductivity on the water content, respectively. By varying each soil particle size fraction on 2% intervals, 1326 unique soils were identified on the textural triangle. The hydrologic responses of each soil were modeled using HYDRUS-1D [34] in response to the three environmental conditions described above. A k-means clustering was then applied to the modeled responses of interest (time-averaged change in water storage in the shallowest 30 cm) to classify the 1326 soils into 12 classes for each of the three environmental conditions. We refer to this direct clustering on hydrologic function as hydrologic-process-based soil textural classification.

Only sand, silt, and clay percentages were considered in this analysis. We inferred hydrauilc properties using the H2 model of ROSETTA, which results in parameters that vary continuously with respect to texture. However, our proposed method allows for inclusion of any other available data, such as bulk density [31], or any other pedotransfer function. It is important to recognize that our proposed hydrologic-process-based soil textural classifications will not be exact. There is uncertainty in the translation of sand, silt, and clay percentages to hydraulic parameters. In our case, we have used a widely accepted pedotransfer function, ROSETTA. But, even this model has uncertainties due to the limited number of samples in the training set. Similarly, numerical flow models are subject to uncertainties due to the equations embedded in the numerical model and in our conceptualization of the flow system. We have used a widely accepted detailed model based on Richards equation (HYDRUS-1D). But, even this model cannot be expected to predict hydrologic outcomes exactly. While our proposed approach is dependent upon the underlying models, it is also able to incorporate any future improvements in these models. The focus of this study is to examine how the underlying concept of process-based soil textures represents an improvement over standard soil textures. For all comparisons presented, both classifications are interpreted through the same pedotransfer function and hydrologic model. A follow on study will quantify how errors in pedotransfer functions propagate through these soil classification schemes.

### Environmental Simulation Flow Conditions

Drainage was simulated using a zero flux top boundary condition, indicating that neither precipitation nor evaporation was occurring. Infiltration at the ground surface was represented by a brief transition from a slightly negative to zero pressure head, producing infiltration at the maximum rate that would not generate runoff. An initial prolonged drainage state in the column was represented by a constant water pressure head of -360 cm throughout the profile; this condition is commonly referred to as field capacity and represents the conditions for which gravity driven drainage approaches zero.

Each condition was modeled for a homogeneous 200 cm column of each soil. The bottom boundary was modeled as free drainage, reducing the impacts of conditions at depth in the target region of 0–30 cm depth. The profile was discretized into 1 cm intervals. Total simulation duration was four days for drainage, one day for infiltration, and five days for infiltration followed by drainage. The time step, however, was allowed to vary in response to the number of iterations needed for convergence in the previous time step (adaptive time stepping). The initial time step was 0.001 seconds. Although adaptive time-stepping was used, results from the simulation were recorded at predefined simulation times to obtain equally spaced model output throughout the simulation period. The reporting time steps were chosen to produce 160 water content profiles during drainage, 40 during infiltration, and 200 for the combined processes.

The data used for clustering was the time-averaged change in length of water stored, $\bar{\Delta L_{w}}$, in the upper 30 cm of the profile. As stated previously and discussed in more detail below, the choice of hydrologic responses can be modified to tune the hydrologically-based soil classification scheme to the conditions and predictions of interest.

### K-means Clustering

Following the approach of Bormann [30], we used clustering based on the k-means algorithm [35] to classify soils based on hydrologic function. Twarakavi et al. [29] and Bormann [30] provide detailed descriptions of k-means clustering; a brief overview is provided here. The k-means clustering algorithm is a centroid-based approach using cluster distortion to organize data points into similar groups [36]. For our application, the data were one-dimensional, defined by the time-averaged change in length of water over a depth of 30 cm for each soil, $\bar{\Delta L_{w}}=\sum_{n=1}^{30}\left(\bar{\Delta \theta_{n}}\right)$. For the conditions representing only infiltration or infiltration followed by drainage, the time averaged change in water content, $\bar{\Delta \theta_{n}}$, is defined as $\bar{\theta_{n}}-\theta_{fc}$, where $\bar{\theta_{n}}$ is the average water content in each layer, and *θ*_*fc*_ is the soil-dependent water content at field capacity. For drainage, $\bar{\theta_{n}}$ is defined as $\theta_{s}-\bar{\theta_{n}}$, where *θ*_*s*_ is the water content of the soil at full saturation, which is equal to the porosity.

Any user-specified prediction of interest could be selected to formulate a soil classification that is most relevant for any specific application. We chose a simple metric: the time averaged water content over the duration of the simulation within the shallowest 30 cm. While the soil parameters, such as *θ*_*fc*_ and *θ*_*s*_, are constant with depth and time, the water content varies both temporally and with depth. The use of the average change in length of water simply allows for clustering on a single outcome, which allows for simpler explanation of the clustering procedure. In fact, there is no limit to the dimensionality of the prediction of interest, ranging from a single value to every value of multiple states through space and time.

With our prediction of interest, infiltration and infiltration followed by drainage would be useful measures of the infiltration capacity (and water holding capacity) of a soil. As such, they may have applicability for predictions related to irrigation timing or slope stability. The definition used for drainage focuses on the ability of a soil to release water after flooding, with applications in trafficability. This range of possible applications demonstrates one of the key attributes of process-based clustering: the high degree of flexibility to define representative soil texture data in ways that are most directly applicable for specific applications.

Clustering is performed for each set of hydrologic process based on $\bar{\Delta L_{w}}$ for all of the soils. The algorithm initially proposes a set of 12 randomly chosen $\bar{\Delta L_{w}}$ values as potential cluster centroids. The $\bar{\Delta L_{w}}$ value calculated for each soil is assigned membership to the closest centroid. Then, the mean of all the $\bar{\Delta L_{w}}$ values for the members in each cluster is calculated to define 12 updated centroid values. The algorithm repeats these steps until no further improvements to the centroids can be made [36]. Improvement or convergence is defined by a reduction in a defined distortion metric. Typically, the metric is defined as the sum of squared distances between the data points and their representative centroid. For our analyses, the k-means algorithm was terminated when the sum of the standard Euclidean, squared normal distances was less than 10^−5^ for whitened data. Since the k-means algorithm does not guarantee a globally minimized distortion, 100 repetitions of the algorithm were performed. The initialization with the lowest final sum of squared distances from the means was selected. Bormann [30] discusses the impacts of distance metrics and numbers of clusters in more detail.

### Cluster Visualization

The hydrologic-process-based soil textural classifications are presented in two forms. First, they are plotted on the USDA soil textural triangle. This provides a basis for direct comparison of the USDA and hydrologic-process-based textural classifications, following the approach of Bormann [30]. This visualization also supports improved understanding of the relationship between particle size distributions and hydraulic behavior. Second, the classifications are used to create revised soil maps for a study site, retrieved from the Natural Resources Conservation Service (NRCS) online database [37]. This visualization is novel to this investigation and underscores the practical and scientific benefits of hydrologic-process-based classification for hypothesis generation.

Python (www.python.org) was used to plot the hydrologic-process-based classifications on the soil textural triangle. Open-source GIS software, QGIS [38], was used to generate maps for visualizing and comparing the hydrologic response for an area north east of Fairfax, OK. Raster data files containing soil unit boundaries and the Microsoft Access database files containing soil texture information were downloaded from the NRCS Web Soil Survey for the study area (S1 Data). The area chosen was 15 × 21 km centered at (36.6121°N, 96.5895°W, mean elevation 311 m) and covers 315 km^2^. The region is representative of the coverage of and variation in soil data that is available from the NRCS Web Soil Survey.

A plugin was developed for creating the soils maps in the open-source QGIS Python based framework (S1 Plugin). It includes a collection of functions for combining and manipulating the spatial and texture data from the NRCS Web Soil Survey. Basic functions were written to update a raster file attribute table to include soil texture data from the Microsoft Access database provided by the NRCS Web Soil Survey. Only the dominant soil texture in the top layer of each soil unit was considered for this study. Other functions were written to match each soil unit to a soil from an existing classification and shade the map units with respect to the assigned cluster in the classification.

## Results and Discussion

The total number of simulations performed was 3978, one for each soil and each set of hydrologic processes. Despite the relatively large number of simulations, the modeling could be completed in 15 minutes on a 64-bit Windows 7 desktop computer with an Intel Core i7 3.47 GHz 6 core processor and 12 GB DDR3 RAM. A similar analysis could be performed for any set of environmental conditions and predictions of interest without unreasonable computational demand. Even though the classifications are hydrologic-process specific, they are still universal with respect to spatial soil texture characteristics. This means that the classifications are independent of the field scale or resolution of soil data and can be applied to any location or hydrologic model if the hydrologic conditions are comparable to those used to define the clusters. In other words, once the hydrologic-process-based classifications are defined, the plugin provided makes it trivial to produce maps for any location with an available soil map.

**Fig 2:**
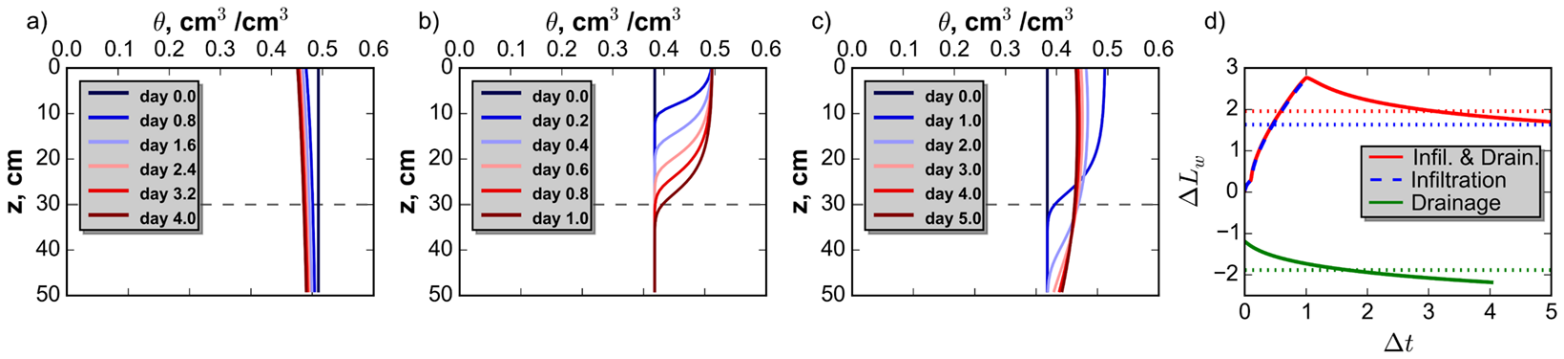
Example Water Content Profiles. Water content profiles through time for a clay soil examined in response to: (a.) drainage, (b.) infiltration, and (c.) infiltration followed by drainage. Horizontal dashed lines show the surface layer depth. (d.) $\bar{\Delta L_{w}}$ for a-c. Horizontal dashed lines show the time-averaged change in *L*_*w*_.

Fig 2a–2c, show soil moisture profiles during each set of hydrologic processes for a clay soil selected from the set of 1326 soils analyzed. A horizontal dashed line shows the depth of the shallow target layer selected for this study. Fig 2d shows the change in length of water in the root zone from the initial condition, as described above, associated with the profiles shown on panels a-c. The time-averaged change in length of water for each set of processes is shown as a dashed horizontal line on Fig 2d. Clustering based on the hydrologic model results required less than a minute of computational time using the computing resources described above.

**Fig 3:**
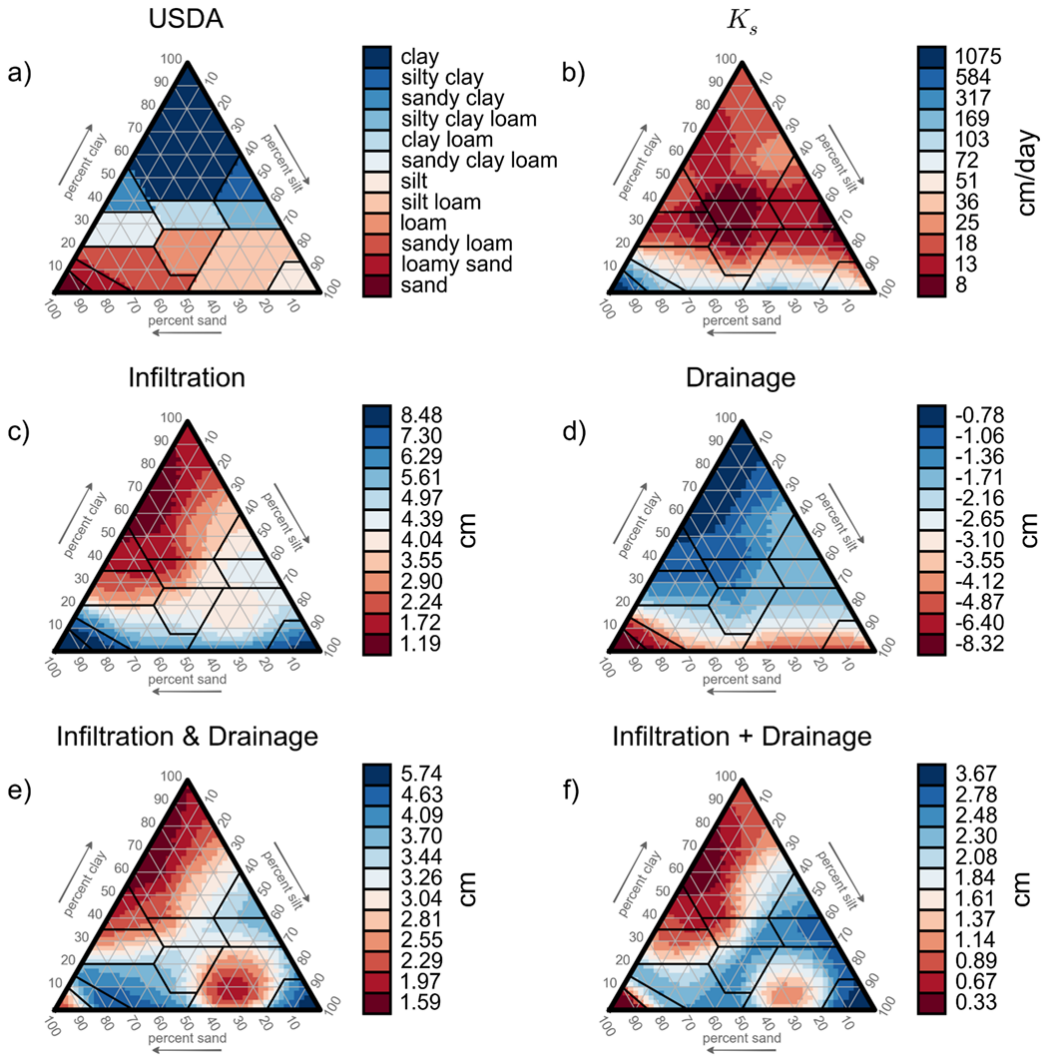
Soil Classifications Plotted on the USDA Texture Triangle. Soil classifications plotted based on (a.) USDA soil classification, (b.) hydraulic conductivity (*K*_*s*_) and $\bar{\Delta L_{w}}$ for: (c.) infiltration, (d.) drainage, (e.) infiltration followed by drainage, and (f.) summation of $\bar{\Delta L_{w}}$ from only infiltration and only drainage. The USDA triangle is shaded by soils texture, the remaining panels are shaded by $\bar{\Delta L_{w}}$ over the simulated period in the uppermost 30 cm.

The USDA soil textural triangle is shown in Fig 3a and classification based on a hydraulic parameter, obtained from ROSETTA, is shown in Fig 3b. The hydrologic-process-based clusters for the three sets of processes considered are shown in Fig 3c–3f. The USDA clusters are defined to be contiguous and to generally coincide with constant values of sand, silt, or clay. These characteristics make it simpler to use USDA clusters for rapid textural mapping in the field. In contrast, the clusters defined based on *K*_*s*_ are irregularly shaped and, in some cases, discontinuous, as are the hydrologic-process-based clusters. While the irregularity and discontinuity of the hydrologic-process-based clusters may make it more difficult to identify clusters from qualitative measures of particle size distributions, the actual classification information shown on the triangle relates directly to the simulated condition. That is, the hydrologically-based clusters are explicitly informed by the simulated hydrologic environmental conditions of each soil. As a result, interpretations of hydrologic response based on these classifications will be inherently more reliable than those based on classifications based on less directly related properties. This reduces the likelihood of misapplication of the cluster information.

General insight can be gleaned from the patterns of hydrologic-process-based clusters. For example, examining the infiltration clusters, Fig 3c, and drainage clusters, Fig 3d, it is apparent that clay-rich soils have low infiltration capacity (red on the infiltration plot, Fig 3c), and low drainage capacity (blue on the drainage plot, Fig 3d). The opposite is true for low-clay soils. The general patterns for infiltration (Fig 3c) and for drainage (Fig 3d) have similar magnitudes, but opposite signs. The magnitude of infiltration and drainage in Fig 3e resulted in low net change in available water in clay-rich soils compared to clay-poor soils. Also, there is a region of low net change in available water for soils with low clay content and approximately equal sand and silt. We also created a classification based on the summation of the separate $\bar{\Delta L_{w}}$ values for infiltration (Fig 3c) and drainage (Fig 3d). This result is referred to as “Infiltration + Drainage” (Fig 3f). Interestingly, the pattern of clustering based on the summation of the infiltration-only and drainage-only results (Fig 3f) shows strong similarities with clustering on infiltration followed by drainage (Fig 3e). These results suggest that it may be possible to cluster soils based on a relatively small number of key sets of hydrologic processes and then to use combinations of these classifications to analyze more complex environmental conditions. This hypothesis is examined further below. Finally, we include Fig 3b. The lack of similarity between classifications based on *K*_*s*_ and those based on the results of modeling hydrologic processes demonstrates that hydraulic conductivity alone is insufficient for inferring hydrologic behavior. No other parameter or set of parameters was found to produce patterns that matched those of the hydrologic-process-based clusters.

The classification patterns produced by Bormann [30] based on hydraulic parameters are similar to the USDA classification and similar to those created by Twarakavi [29], which were also based on hydraulic parameters and characteristics. However, the simple polygons produced by those classifications differ from those shown here based on hydrologic responses, which have patterns that are much more irregular and discontinuous. Bormann [30] produced classifications based on mean annual water balance; these too are different than the classifications shown here based on hydrologic response in terms of change in length of water. Assuming that soil textures will be used for hydrologic prediction, the lack of agreement between the classifications based on hydraulic parameters or texture with those based directly on hydrologic response demonstrates that other approaches to soil classification will not result in optimal interpretation of hydrologic function.

Twarakavi, et. al. [29], demonstrated that specific collections of parameters and soil characteristics can be chosen that reflect classification patterns similar to the USDA soil texture classification. However, we contend that it is impossible to define a single set of parameters that will lead to clusters that are relevant for a range of hydrologic predictions. Simply stated, some processes depend more strongly on storage capacity, others on permeability, and still others on a more equal combination of the two. Furthermore, for any given soil, both storage capacity and permeability depend on the water content. Therefore, each of these parameters depends on both the average flux through the system and the variation in flux through time. Our simple premise is that the best way to determine how different soils will behave under a set of applied conditions is to model the responses and then redefine the soils based on those hydrologic outcomes.

**Fig 4:**
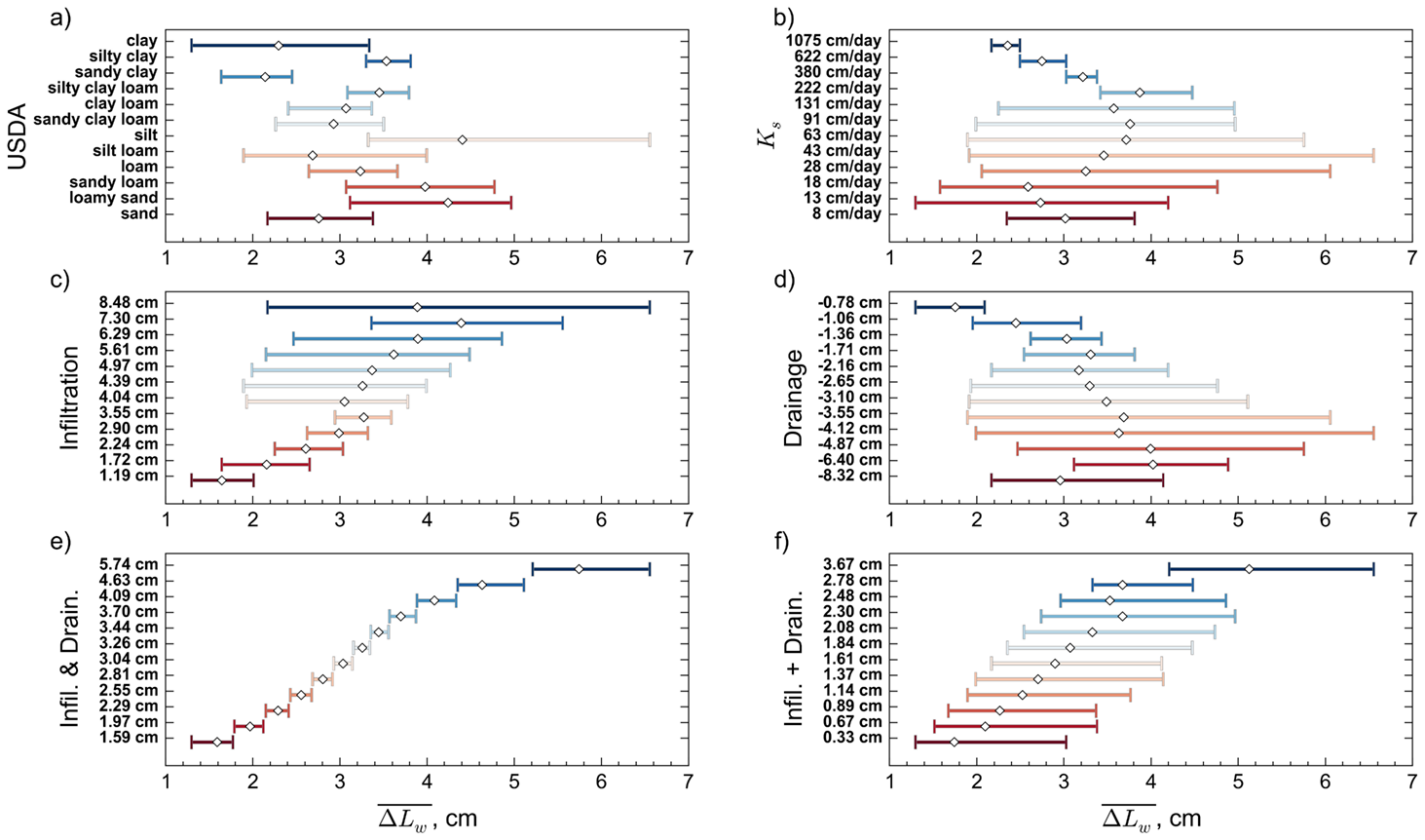
Predictive Effectiveness for each Classification. Time averaged $\bar{\Delta L_{w}}$ values within each cluster. The symbol shows the mean value and the bars represent minimum and maximum values within the cluster. Classifications are labeled as in Fig 3

To compare the performance of the USDA and hydrologic-process-based clusters, we make a simple proposal: the utility of a clustering scheme can be assessed by the uniqueness of predictions among clusters. That is, an effective scheme will have predictions of interest that are more similar within a cluster than between clusters. Fig 4 demonstrates this test in terms of the skill of predicting the change in $\bar{\Delta L_{w}}$ to a depth of 30 cm for the third environmental condition having one day of infiltration followed by four days of drainage. Each panel shows the range of predictions (shown as a bar) and the mean prediction (shown as a symbol) for each cluster. The vertical axis is labeled using the cluster centroid from Fig 3, except for the USDA texture triangle (panel a) where the soil name is used. The mean prediction is identical to the centroid for the cluster-based results in Fig 4e. In addition, we also show the results of clustering on *K*_*s*_ to allow for comparison with parameter-based clustering such as shown by Twarakavi et al. [29].

The ultimate practical objective of soil mapping for hydrologic applications is to provide an unambiguous and easily interpreted representation of the spatial distribution of the hydrologic process or processes that are important for a given application. We contend that this is best achieved by clustering as closely as possible on the hydrologic outcome as calculated using a process model and the use of a pedotransfer function. In our synthetic case, with perfect information about mapping sand, silt, and clay to hydraulic properties and perfect knowledge of the forcings, we can generate clusters with no overlap (no ambiguity). This is shown in Fig 4e. We do not mean to imply that the lack of overlap indicates that hydrologic-process-based classification results in perfect predictions. Rather, the lack of overlap in Fig 4e should be seen as the theoretically optimal outcome. Deviations from this unambiguous clustering demonstrate limitations of other clustering approaches. It should be reiterated that all analyses presented here benefit form perfect mapping of soil properties onto sand, silt, and clay fractions and correct hydrologic model structures and forcings. The propagation of uncertainties in the underlying pedotransfer function through process-based clustering will be the subject of a follow on study.

Clustering on $\bar{\Delta L_{w}}$ for infiltration and drainage (Fig 4e) produces predictions with little to no overlap among clusters. That is, each cluster has a unique meaning with respect to the prediction of interest. This is one of the benefits of and motivations for hydrologic-process-based clustering. In stark contrast, the USDA soil textural classification (Fig 4a) shows a high degree of overlap in prediction ranges. Furthermore, there is no clear relationship between the mean and the order of clusters or textures as typically listed from sand to clay. Fig 4c and 4d show the ability of hydrologic-process-based clustering when the basis for clustering is different than the processes being predicted. Although general trends appear across the means of the clusters, they still lack the degree of uniqueness that is present in Fig 4e. This suggests that a single hydrologic-process-based classification may not be sufficient for defining hydrologically relevant soil textures across a wide range of hydrologic processes: hydrologic-process-based soil textures are not universal, they are application specific. Finally, we examine the quality of predictions based on clustering on the summation of the $\bar{\Delta L_{w}}$ for infiltration and drainage, as shown in Fig 3f. In this case, the trend in increasing net change in available water is reasonably well represented, but the results lack the uniqueness of the classification based on infiltration and drainage (Fig 3e). The balance between the improved uniqueness of predictions offered by more process-specific clusters with the reduced effort offered by combining the clusters from a sequence of single processes will also be a topic of future research.

In hydrologic sciences and atmospheric sciences, soil hydraulic properties are often considered the most useful properties for classifying soils. To examine this assumption, we considered clustering based on the saturated hydraulic conductivity, *K*_*s*_ (Fig 3b), which is a fundamental measure of the ease with which water can move through a soil under water saturated conditions. Interestingly, this clustering shows even less skill than the USDA classification. In particular, many of the *K*_*s*_-based clusters have very large within-cluster variations. In addition, there is no clear correlation between increasing *K*_*s*_ and increasing $\bar{\Delta L_{w}}$. This supports the conclusion by Norris [39] that generalization of soils based on only a few parameters is not advisable. Norris suggests that different generalizations would result for comparisons made between single properties because of high parameter interaction. It may be surprising that this key hydraulic property (*K*_*s*_) fails to provide information about hydrologic processes. This highlights the complex interactions among multiple soil properties and dependence on boundary and initial conditions that make direct inferences based soil hydraulic properties challenging. In other words, model simulations are able to reliably capture behaviors that parameters alone cannot. Even consideration of multiple hydraulic properties is unlikely to improve on the USDA classifications, given that Twarakavi et al. [29] demonstrated that clusters based on collective soil properties are comparable to the USDA classification, which do a poor job of predicting hydrologic outcomes (Fig 4a). We contend that this result is a direct indication of improvement in predictive skill that can be achieved by using clustering based on hydrologic processes rather than on hydraulic properties or by using standard soil textures.

To evaluate the skill of prediction for the different classification schemes more quantitatively, we considered several defining characteristics. First, we examined the trend of the cluster means with the cluster order (Fig 4). As Fig 4e shows, infiltration followed by drainage has the smoothest increasing trend behavior and has a strong correlation between the prediction centroid and the actual $\bar{\Delta L_{w}}$. This trend highlights the continuous pattern of clusters in Fig 3e and is also present in Fig 3c–3d. This is followed closely by the addition of infiltration and drainage. Although the means for infiltration alone and drainage alone show a general trend, the trend is not as consistent as seen for clusters that consider both infiltration and drainage. The USDA classification and hydraulic conductivity-based clustering show very poor trends with cluster order. (It is difficult to quantify the trend for the USDA classification because the order of the classes is based on descriptive labels rather than a numerical value, but the trend is clearly discontinuous with the soil textures in the order commonly presented).

To provide more quantitative performance measures of the clustering approaches, we examine the uniqueness of the correlation of the prediction of interest with the clusters (and vice versa). First, we evaluate the performance of the classifications based on the sum of squared distances of the cluster members from their centroids. This calculation is similar to the convergence criteria for the k-means algorithm. Next we consider the uniqueness with which soil members map onto the clusters. In this case, the uniqueness is based on the change in length of water per unit area. That is, how many different clusters could be associated with each value of $\bar{\Delta L_{w}}$? We refer to this as outcome-cluster uniqueness, or OC uniqueness. The average value of this metric across all clusters ranges from 1 (best possible performance, all outcomes values are unique to single clusters) to the total number of clusters (12 in our case, meaning that every cluster spans all possible outcomes). Lastly, given the range of $\bar{\Delta L_{w}}$ values included within a cluster, what fraction of the soil members that fall within this range are not members of that cluster? We refer to this as cluster range uniqueness, or CR uniqueness. The average value over all of the clusters ranges from 0 (best possible performance) to 1. Table 1 summarizes the results for the clustering approaches presented above and ranks the performance of the metrics from 1 (best performance) to 6 (poorest performance).

**Table 1:** Performance Metrics When Predicting Infiltration & Drainage.

	**RMSE**	**rank**	**OC**	**rank**	**CR**	**rank**
**I & D**	0.12	1	1.0	1	0.00	1
**I + D**	0.41	3	7.0	4	0.84	5
**USDA**	0.37	2	5.5	2	0.83	4
***K*_*s*_**	0.60	6	8.3	6	0.90	6
**I**	0.47	4	6.5	3	0.79	2
**D**	0.58	5	7.7	5	0.80	3


OC is outcome-cluster uniqueness and CR is cluster-range uniqueness.

These results (Table 1) show that application specific clustering (Infiltration followed by Drainage, I & D) is clearly superior, ranking first in all three categories. All of the other approaches have uniqueness measures that are very close to one another (within: 0.23 for RMSE, 2.8 for OC and 0.11 for CR) and also perform poorly (greater than: 0.37 for RMSE, 5.5 for OC and 0.79 for CR). To put these performance metric values in context, they suggest that it is at least as likely that any given outcome will be miscategorized as correctly categorized. Clusters based on drainage alone or infiltration alone result in clusters of infiltration and drainage predictions that are more distinct than hydraulic conductivity classification. Interestingly, even though the visual pattern of clusters based on the addition of infiltration and drainage is very similar to clustering directly on infiltration followed by drainage, I + D does not perform well when assessed quantitatively. It is also interesting to note that the USDA clustering is, arguably, as good or better than any of the clustering approaches except for the clearly superior I&D. The superiority of clusters based on the specific hydrologic processes of interest, together with the fact that the clustering analysis can be performed with relatively little computational effort, suggests that classifications should be as process-specific as possible.

**Fig 5:**
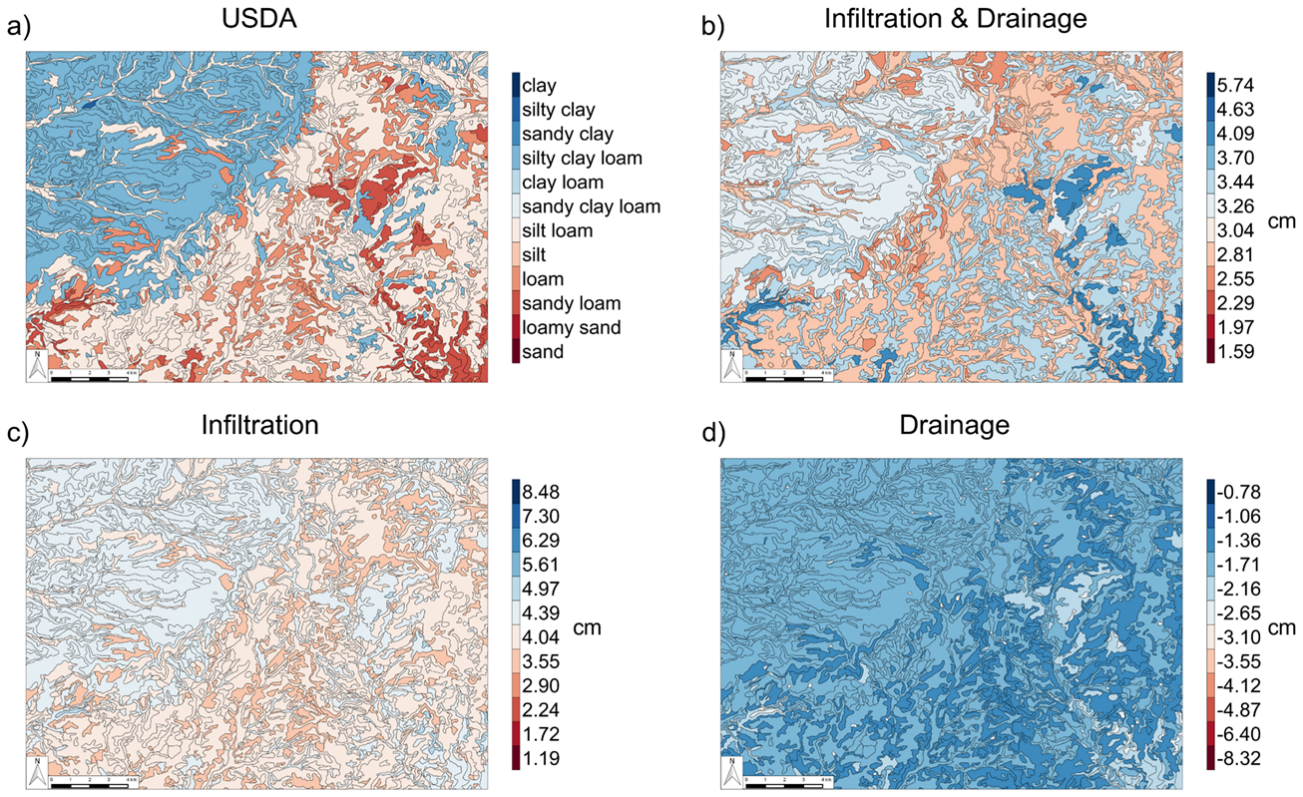
Classification Soil Maps. Soil maps based on (a.) USDA soil classification; and hydrologic-process-based classifications based on $\bar{\Delta L_{w}}$ (b.) for infiltration & drainage; (c.) infiltration and, (d.) drainage.

In practice, soils are categorized for many purposes, including vehicle trafficability; vegetative productivity; erodibility; dust generation capacity; and flood susceptibility. Given that the spatial patterns of soils on a landscape impact many of these processes, it is valuable to produce maps that show the spatial organization of soil function [25]. Fig 5 shows four different visualizations of soil hydrologic function.

In Fig 5a, the landscape is represented using the USDA soil textural classification. Soil maps like this are widely available and used commonly to characterize agricultural soils at field and watershed scales. The question that we aimed to address is: can a map based on soil texture help to generate useful hypotheses about patterns of hydrologic processes across a landscape? Further, can more insight be gained by applying hydrologic-process-based classifications? The results of clustering on hydrologic processes are shown in Fig 5b–5d. Fig 5c shows that there is very little variation (3.55–4.97 cm) in infiltration capacity across the landscape compared to the range of possible values across the I&D soil types (1.19 cm to 8.48 cm). Similarly, there are relatively few areas that show significant drainage (Fig 5d); in general, drainage rates are low over the entire site, below -2.65 cm, with very low rates in the southeastern part of the domain.

Finally, the net increase in water storage (dark blue on Fig 5b) after a cycle of infiltration and drainage is limited to a relatively small region in the southeast corner of the domain. There is also a band of moderately low net infiltration (light red) that trends from the northeast to the southwest across the domain. Given the variance in predictions of infiltration and drainage within the USDA clusters (Fig 4e), both of these features would be difficult if not impossible to identify from the USDA map, even for someone who is very familiar with the hydraulic properties of different soils. In contrast, important patterns are immediately apparent on the hydrologic-process-based soil maps. For example, following a flooding event, Fig 5d would clearly identify areas that are likely to be slow to drain, leading to low trafficability for emergency or agricultural equipment. Fig 5c, on the other hand, predicts areas that may be good candidates for intentional routing of water for rapid infiltration during intense rainstorms. Lastly, plant community establishment efforts could benefit from understanding the spatial patterns of water holding capacity of soils as described by Fig 5b.

**Fig 6:**
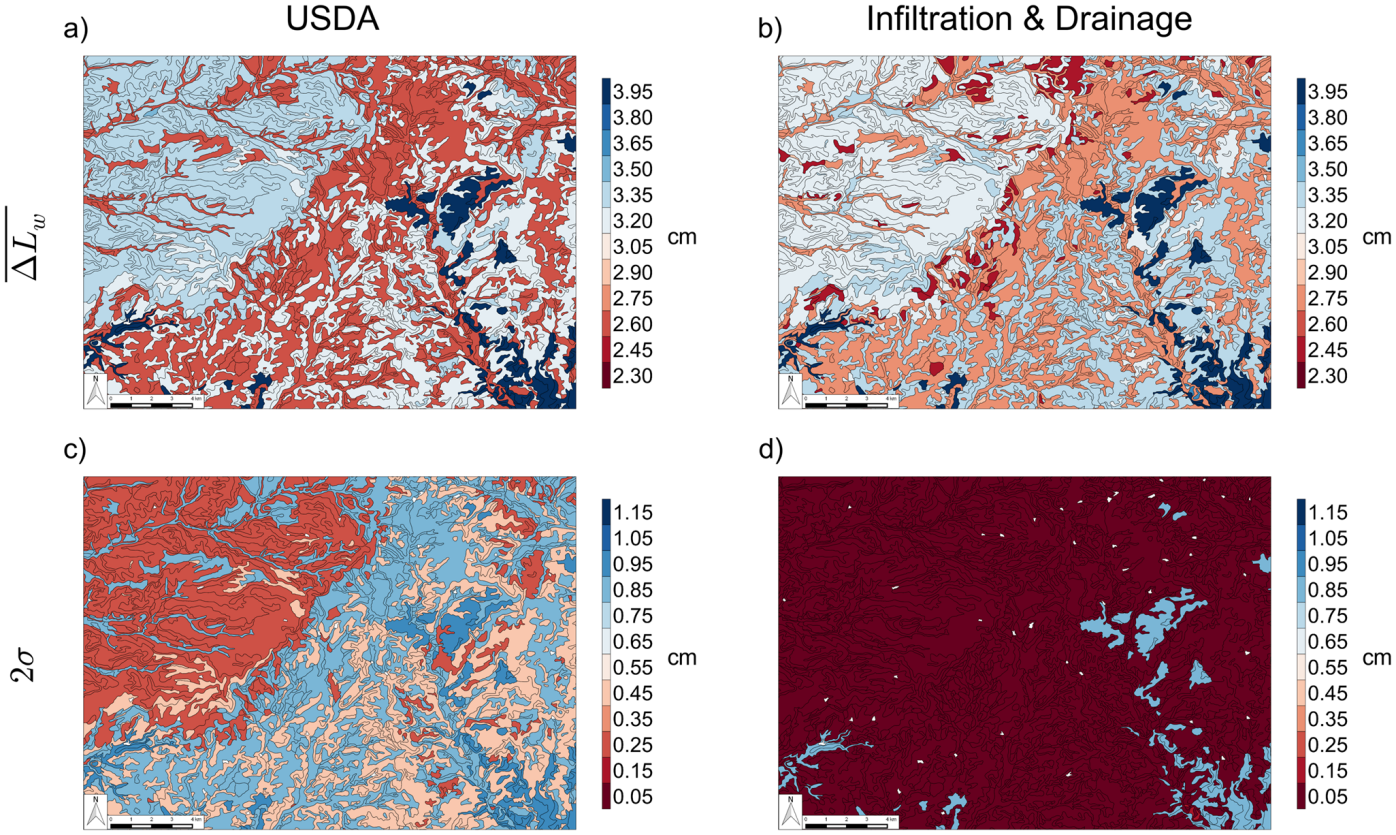
Soil Map Prediction Variation. Soil maps showing the range of $\bar{\Delta L_{w}}$ following infiltration and drainage based on the classification of (a.) USDA soil texture and (b.) infiltration and drainage. Soil maps showing two standard deviations of the values of change in length of water following infiltration and drainage based on the classification of (c.) USDA soil texture and (d.) infiltration and drainage.

While Fig 5 shows the expected hydrologic response on different soil texture definitions, this does not present a complete measure of the utility of these hydrologic function maps. It is also necessary to understand the level of uncertainty of the results shown. The panels in Fig 6 show the predicted infiltration and drainage maps based on the USDA classification (first column) and a classification based on infiltration and drainage (second column). These maps are created from the data shown in Fig 5. The mean value of the change in length of water for the cluster (texture- or process-based) is shown for each soil feature on the maps in the top row of Fig 6. For the bottom row in Fig 6, two standard deviations of the change in length of water within the cluster is shown. For clarity, the color scale is limited to the range of values seen on both maps. Both approaches (USDA clustering and hydrologic-process-based clustering) show considerable variability in the hydrologic response, making it difficult to choose which is more useful. However, the uncertainty of the predictions due solely to the classification approach (both approaches use the identical pedotransfer function and hydrologic model) is considerably higher through most of the domain for the USDA classifications. This is our main finding: the use of hydrologic-process-based classification can produce less ambiguous, more practically useful plots that can be used more quantitatively by experts and interpreted reliably by non-experts.

In summary, we suggest that models should be run for a very wide range of soils for the expected forcings (those that represent the expected hydrologic conditions at a given location that are most relevant for a specific application of interest). Then, soil “types” should be defined based on outcomes of the models rather than on sand, silt, and clay percentages, other physical properties, or hydraulic parameters. The resulting maps, will be less ambiguous and more readily interpreted than soil texture maps.

## Conclusions

The objective of this work was to examine the effectiveness of soil textural based classifications at capturing hydrologic response and to consider an alternative approach to classification that could capture hydrologic response. We have shown that soil-texture-based (e.g. USDA) and parameter-based [29] classifications do not provide accurate predictions of responses to hydrologic conditions. Specifically, the patterns of clustering on the soil texture triangle, quantitative measures of cluster effectiveness, and maps produced to improve understanding of soil function on the landscape perform poorly when based on the USDA textures or the saturated hydraulic conductivity. Our results suggest that greater insight into hydrologic behavior will result from clusters based directly on relevant hydrologic processes.

Our analysis is based on a simple set of common hydrologic conditions. We examined whether these basic hydrologic processes could be combined rather than clustering on a specific boundary condition time series. While the resulting clusters are visually similar, quantitative measures of cluster performance suggest that combining data from individual and separate processes is not as accurate as creating application-specific clusters for sets of relevant hydrologic forcings. The Natural Resource Conservation Service itself has long acknowledged that classifications were designed for specific uses and that it should not only be possible to construct libraries of classifications as the need arises, especially as technology and knowledge allows [29]. Our results amplify this finding, suggesting that no globally optimal soil textural classification is likely to exist. For this reason and because minimal computational time and effort are required, we suggest that hydrologic-process-based clusters be calculated to form application-specific soil texture classifications.

Our work has confirmed the conclusions reached by Bormann [30] and has shown how their approach can be extended to a wide range of applications that rely on prediction of hydrologic response. In particular, echoing the finding of Bormann [30], we find that there is clear benefit in defining soil “types” based on application-specific hydrologic responses. This would lead to a new concept of soil type—one that is not universal across all applications, but rather, is based on a common framework for application-specific definition.

We suggest that the use of soil maps to generate hypotheses of soil function across a landscape is greatly hindered by the use of USDA soil textures. This is supported by the extensive differences in texture maps based on hydraulic function compared to those based on soil texture and the large uncertainties associated with texture-base hydrologic-function maps. Hydrologic-process-based classifications produce maps that are easily interpreted, lacking the errors and ambiguity that would result from inference of landscape function based on the USDA classifications. While we have focused on hydrologic applications, the underlying concept of process-specific clustering presented could be applied using any set of predictions of interest with corresponding geospatially referenced data and appropriate pedotransfer functions.

## Supporting Information

S1 Data NRCS WSS AOI data. Spatial, tabular and soil database files for the Oklahoma region that was used for creating the soil maps downloaded from the NRCS Web Soil Survey.
(ZIP)

S1 Plugin QGIS Classification Visualization Plugin. Plugin that was written for creating the soil maps using data from the NRCS Web Soil Survey.
(ZIP)

S2 Data Classification Data. Observations of average change in length of water and *K*_*s*_ used to create the classifications with corresponding sand, silt and clay fractions.
(XLSX)
